# Central Nervous System PET-CT Imaging Reveals Regional Impairments in Pediatric Patients with Wolfram Syndrome

**DOI:** 10.1371/journal.pone.0115605

**Published:** 2014-12-26

**Authors:** Agnieszka Zmyslowska, Bogdan Malkowski, Wojciech Fendler, Maciej Borowiec, Karolina Antosik, Piotr Gnys, Dobromila Baranska, Wojciech Mlynarski

**Affiliations:** 1 Department of Pediatrics, Oncology, Hematology and Diabetology, Medical University of Lodz, Lodz, Poland; 2 Department of Nuclear Medicine, Oncology Center, Bydgoszcz, Poland; 3 Department of Clinical Genetics, Medical University of Lodz, Lodz, Poland; 4 Department of Radiology, Clinical Hospital No 4, Lodz, Poland; University of Jaén, Spain

## Abstract

Wolfram syndrome (WFS) is inherited as an autosomal recessive disease with main clinical features of diabetes mellitus, optic atrophy, diabetes insipidus and deafness. However, various neurological defects may also be detected. The aim of this study was to evaluate aspects of brain structure and function using PET-CT (positron emission tomography and computed tomography) and MRI (magnetic resonance imaging) in pediatric patients with WFS. Regional changes in brain glucose metabolism were measured using standardized uptake values (SUVs) based on images of (^18^F) fluorodeoxyglucose (FDG) uptake in 7 WFS patients aged 10.1–16.0 years (mean 12.9±2.4) and in 20 healthy children aged 3–17.9 years (mean 12.8±4.1). In all patients the diagnosis of WFS was confirmed by DNA sequencing of the *WFS1* gene. Hierarchical clustering showed remarkable similarities of glucose uptake patterns among WFS patients and their differences from the control group. SUV data were subsequently standardized for age groups <13 years old and>13 years old to account for developmental differences. Reduced SUVs in WFS patients as compared to the control group for the bilateral brain regions such as occipital lobe (−1.24±1.20 *vs.* −0.13±1.05; p = 0.028) and cerebellum (−1.11±0.69 *vs.* −0.204±1.00; p = 0.036) were observed and the same tendency for cingulate (−1.13±1.05 *vs.* −0.15±1.12; p = 0.056), temporal lobe (−1.10±0.98 *vs.* −0.15±1.10; p = 0.057), parietal lobe (−1.06±1.20 *vs.* −0.08±1.08; p = 0.058), central region (−1.01±1.04 *vs.* −0.09±1.06; p = 0.060), basal ganglia (−1.05±0.74 *vs.* −0.20±1.07; p = 0.066) and mesial temporal lobe (−1.06±0.82 *vs.* −0.26±1.08; p = 0.087) was also noticed. After adjusting for multiple hypothesis testing, the differences in glucose uptake were non-significant. For the first time, regional differences in brain glucose metabolism among patients with WFS were shown using PET-CT imaging.

## Introduction

Wolfram syndrome (WFS) (OMIM 222300) is inherited as an autosomal recessive trait. Wolframin, the product of the *WFS1* gene, is an integral component of the endoplasmic reticulum (ER) and protects cells from ER stress, which occurs due to accumulation of endogenously synthesized protein products. Loss of wolframin function in particular cells thus gives rise to greater ER stress, resulting in apoptosis of the affected cells [1], [2]. The clinical criteria for WFS are diabetes mellitus and optic atrophy coexisting with other disorders, especially neurodegenerative ones. Other symptoms include progressive deafness, diabetes insipidus, and urodynamic and neurological abnormalities [3]. One of the most frequent neurological presentations is cerebellar ataxia with gait disturbances. In addition, cognitive impairment, nystagmus, anosmia, lack of tendon reflexes in the extremities, epilepsy, dysphagia, polyneuropathy, and central sleep apnea are also observed [3], [4]. Neurological complications are the most common cause of death in WFS patients, who have an average life expectancy of about 30 years [5], [6], [7]. However, discrepancies between the degree of clinical manifestation of neurological disorders and the results of imaging studies of the central nervous system have been noted in some WFS patients [8], [9]. Moreover, attempts to find a genotype-phenotype correlation between the severity of neurological defects and the type of *WFS1* mutation have been unsuccessful.

Neuroimaging studies of WFS patients show brain atrophy mainly affecting the optic nerves, cerebellum, hypothalamus, hippocampus, pons and brain stem, but there is also evidence of generalized brain atrophy based on magnetic resonance imaging (MRI) [10], [11], [12], [13]. It is known that positron emission tomography and computed tomography (PET-CT), and recently available PET-MRI, gives more sensitive and specific brain assessment in comparison with other available neuroimaging methods in patients with neurodegenerative diseases [14], [15]. Thus, the aim of the study was to evaluate aspects of the structure and function of the brain in WFS pediatric patients versus healthy control subjects by using PET-CT and MRI.

## Materials and Methods

Wojciech Mlynarski is the guarantor of the study and, as such, had full access to all the data in the study and takes responsibility for the integrity of the data and the accuracy of the data analysis. The raw data will be made available from the authors on request.

The Ethics Committee of the Medical University of Lodz approved the project (RNN/140/13/KE). Parents gave written informed consent for participation of their children in the study.

The study group comprised 7 females with WFS aged 10.1–16.0 years (average 12.9±2.4). The control group consisted of 20 healthy non-diabetic children (14 F/6 M) aged 3–17.9 years (average 12.8±4.1). Diagnosis of WFS was confirmed by DNA sequencing of the *WFS1* gene, as described previously [16]. Six patients had diabetes and optic atrophy. One patient (WFS7), homozygous for the W540X mutation, had diabetes mellitus without optic atrophy. Patients WFS1 and WFS2, WFS3 and WFS4, and WFS6 and WFS7 are siblings. In all patients with WFS a typical neurological examination was performed with an evaluation of the skull, cranial nerves, motor system (posture and muscle strength), cerebellar function (coordination) and reflexes.

Mean diabetes duration of the study group was 7.5±4.1 years. Mean HbA1c level was 7.48±0.95%. Detailed clinical and genetic characteristics of the children with WFS are shown in Table 1.

**Table 1:** Clinical and genetic characteristics of patients with Wolfram syndrome (16, 30).

Patient ID	Diabetes Mellitus (age at diagnosis)	Optic atrophy (age at diagnosis)	Diabetes insipidus	Deafness	Renal tract disorders	Neurological disorders	Diabetes duration (years)	Mutation (nucleotide change)	Mutation (amino acid change)
WFS1	4	5	Yes	No	Yes	Yes	5	c. 1329C>G#	Homozygous p.S443R#
WFS2	5	5.5	Yes	No	Yes	Yes	12	c. 1329C>G#	Homozygous p.S443R#
WFS3	4	6	Yes	Yes	No	Yes	7	c. 1619G>A#	Homozygous p.W540X#
WFS4	5	9	No	No	No	Yes	9	c. 1619G>A#	Homozygous p.W540X#
WFS5	8.7	9.7	No	Yes	Yes	Yes	5	c. 501delC/c. 1943G>A* #	p.S167E/p.W648X* #
WFS6	6	10	Yes	Yes	No	Yes	13	c. 1619G>A##	Homozygous p.W540X##
WFS7	4.5	No	No	No	No	No	1.5	c. 1619G>A##	Homozygous p.W540X##

*according to Human Gene Mutation Database; accession No: CM 982041.

# a patient with a first description in [16].

## a patient with a first description in [30].

### Image acquisition

Regional changes in brain glucose metabolism were measured using standardized uptake value (SUV) to analyze images of (^18^F) fluorodeoxyglucose (FDG) uptake. For FDG-PET-CT examinations, 5–6 MBq/kg of (^18^F) FDG [maximum 12 mCi (444 MBq)] was injected intravenously into patients after fasting period of at least 6 h. Blood glucose was determined to be normal prior to injection and was in the range 110–140 mg/dl. Patients did not receive sedation for examinations. The patients stayed in a quiet, dark room after the injection and were encouraged to remain recumbent and relaxed for at least 15 min before examination. Imaging was performed using Biograph 16 and Biograph mCT 128 scanners (Siemens, Germany). In 4 patients with WFS an acquisition was performed using Biograph mCT 128 and in 3 cases using Biograph 16. In the control group 9 children were studied with Biograph mCT 128 and 11 children with Biograph 16.

Transmission CT images for attenuation correction and PET images were acquired approximately 30 min after injection of the tracer.

CT acquisition parameters were as follows:

- Biograph 16: slice thickness of 0.5 cm, tube rotation of 0.6 s, pitch of 1.5, 130 kV, mAs-CARE Dose 4D

- Biograph mCT128: slice thickness of 0.3 cm, tube rotation of 0.5 s, pitch of 0.8, 120 kV, mAs-CARE Dose 4D

PET images of the brain were obtained by 20 min acquisition on each scanner. A head holder stabilized the patient's head. Images were reconstructed by using standard vendor-supplied software (Iterative3D-Biograph16, TrueX+TOF-mCt128).

Head PET images were normalized using Scenium Ratio Analysis software (Siemens Medical Solutions, USA), a probabilistic population-based brain atlas of human cortical structures. Spatial normalization was performed by an automated image registration toolkit, which used the ratio of image uniformity as the objective function. The agreement of spatial normalization was evaluated qualitatively by visual inspection of normalized PET. Due to the lack of representation of the brain stem in standard Scenium areas, the brain stem was determined manually by placing a volumetric VOI on the brain stem volume visible in the CT scan.

The obtained SUV results were analyzed for separate small right-side and left-side areas of the brain and for bilateral average values, and then for a small number of right-side and left-side main fields of the brain and their bilateral average values.

In addition, MRI data were available for the WFS patients, collected on average 14 months (range 0.5 to 18 months) prior to the FDG-PET-CT study. In each subject a full standardized brain MRI protocol consisting of the sequences in T1-weighted, T2-weighted and FLAIR (fluid attenuated inversion recovery) MR images in axial, coronal and sagittal sections were performed making a number of about 300 scans for each patient with WFS. Two experienced radiologists evaluated all images independently. Observed lesions were measured in three dimensions: AP (anterior-posterior), H (height) and T (transverse) in millimeters (mm). As an optic nerve atrophy we defined a thinning of the nerve less than 4–5 mm in diameter with no signal change. Optic nerves were assessed by measuring a transverse diameter in the intracranial and intraorbital parts of nerves in axial, coronal and sagittal scans on T2-weighted MR sequences.

### Statistical analysis

Hierarchical clustering was performed on SUVs by using Euclidean distances to evaluate within-group similarities. Statistical comparisons were performed on raw data and after standardization of SUVs for two age groups of patients. Average values from corresponding regions of interest (ROIs) were calculated in every patient, since SUVs observed in the left and right hemispheres were convergent across all individuals (S1 Table). Data of 10 healthy control subjects younger than the 13 years were used as the reference standard for the younger group, and data of the other 10 healthy children older than 13 years were used for standardization of data from the older patients. For all ROIs mean and standard deviation from both older and younger healthy groups were computed and, subsequently, the data of all individuals were standardized accordingly.

In this way we were able to account for developmental differences in the absence of an otherwise eligible reference group of age-matched controls. Student's t-test was used for comparison of patients with WFS and healthy individuals. To control for type 1 errors, we used the Benjamini-Hochberg adjustment for multiple hypothesis testing. The threshold for declaring significance was an adjusted p value lower than <0.05. Analyses were performed using Statistica 10.0 PL software (Statsoft, Tulsa, OK, USA).

## Results

Diabetes and optic atrophy were present in all WFS patients except one (WFS7) with diabetes only. Neurological signs were observed in all but one patient (WFS7), and included the following: ataxia/gait disturbances (WFS1, WFS2, WFS3, WFS4, WFS6), nystagmus (WFS1, WFS3, WFS6), lack of tendon reflexes in the extremities or hyporeflexia (WFS3, WFS4, WFS5), cognitive disorders (WFS2, WFS4, WFS5), dysphagia (WFS3), and polyneuropathy (WFS1) (Table 2).

**Table 2:** Neurological characteristics and detected changes in brain MRI and PET-CT studies in patients with Wolfram syndrome.

Patient ID	Neurological signs	Changes on brain MRI	Regions of brain with changes visible on PET-CT
WFS1	Optic atrophy, ataxia, polyneuropathy, nystagmus	Atrophy of optic nerves, chiasm and tracts	Cerebellum, mesial temporal lobe
WFS2	Optic atrophy, ataxia, cognitive disorders	Atrophy of optic nerves, chiasm and tracts	Cerebellum, cingulate, occipital lobe, temporal lobe, mesial temporal lobe
WFS3	Optic atrophy, ataxia, nystagmus, lack of tendon reflexes in the extremities, dysphagia	Atrophy of optic nerves, chiasm and tracts	Cerebellum, occipital lobe, basal ganglia, central region, parietal lobe, temporal lobe
WFS4	Optic atrophy, ataxia, hyporeflexia, cognitive disorders	Atrophy of optic nerves, chiasm and tracts	Cerebellum, cingulate, occipital lobe, basal ganglia, central region, parietal lobe, temporal lobe
WFS5	Optic atrophy, hyporeflexia, cognitive disorders	Atrophy of optic nerves, chiasm and tracts	Cerebellum, cingulate, occipital lobe, basal ganglia, central region, parietal lobe, temporal lobe
WFS6	Optic atrophy, gait disturbances, nystagmus	Atrophy of optic nerves, chiasm, tracts and bilateral multiple atrophy of brain stem (<16-mm lesions) and in the paraventricular white matter	Cerebellum, basal ganglia, central region, occipital lobe, hippocampus, temporal lobe, thalamus
WFS7	None	No information	Cerebellum, basal ganglia, occipital lobe, hippocampus, thalamus

**Figure 1:**
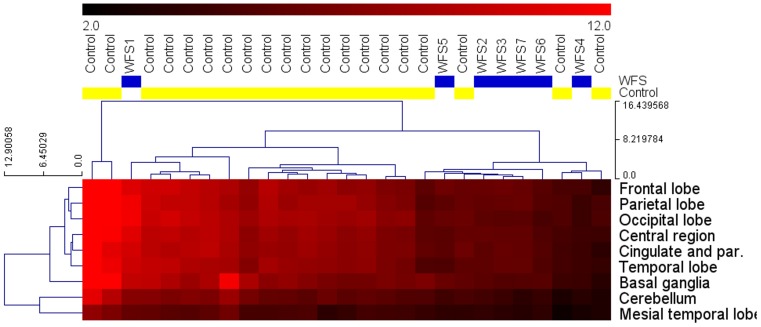
Signal intensity profiles in particular areas of the brain based on hierarchical clustering analysis. The red bar above the chart represents SUV values observed in the examined individuals.

**Figure 2:**
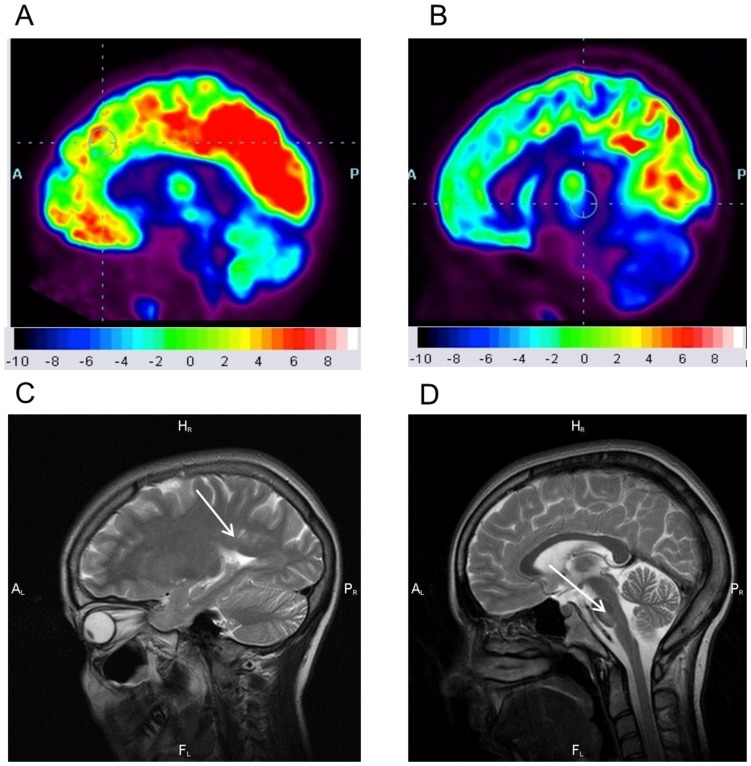
Differences in FDG uptake in PET-CT scans of the brain between a healthy child (a) and WFS6 patient (b) and small lesions in MRI scans in WFS6 patient in the paraventricular white matter (c) and brain stem (d).

**Figure 3:**
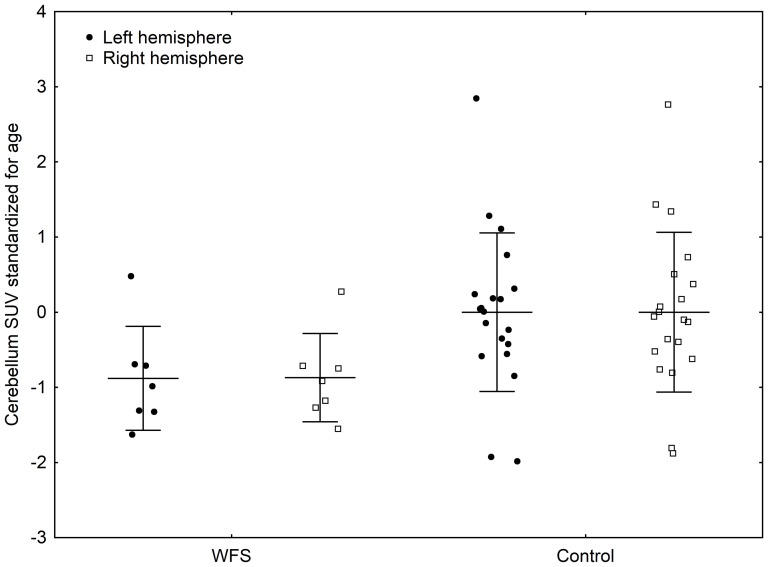
Mean SUV and SD values (standardized for age) for the right and left cerebellum hemispheres in WFS and control groups.

**Figure 4:**
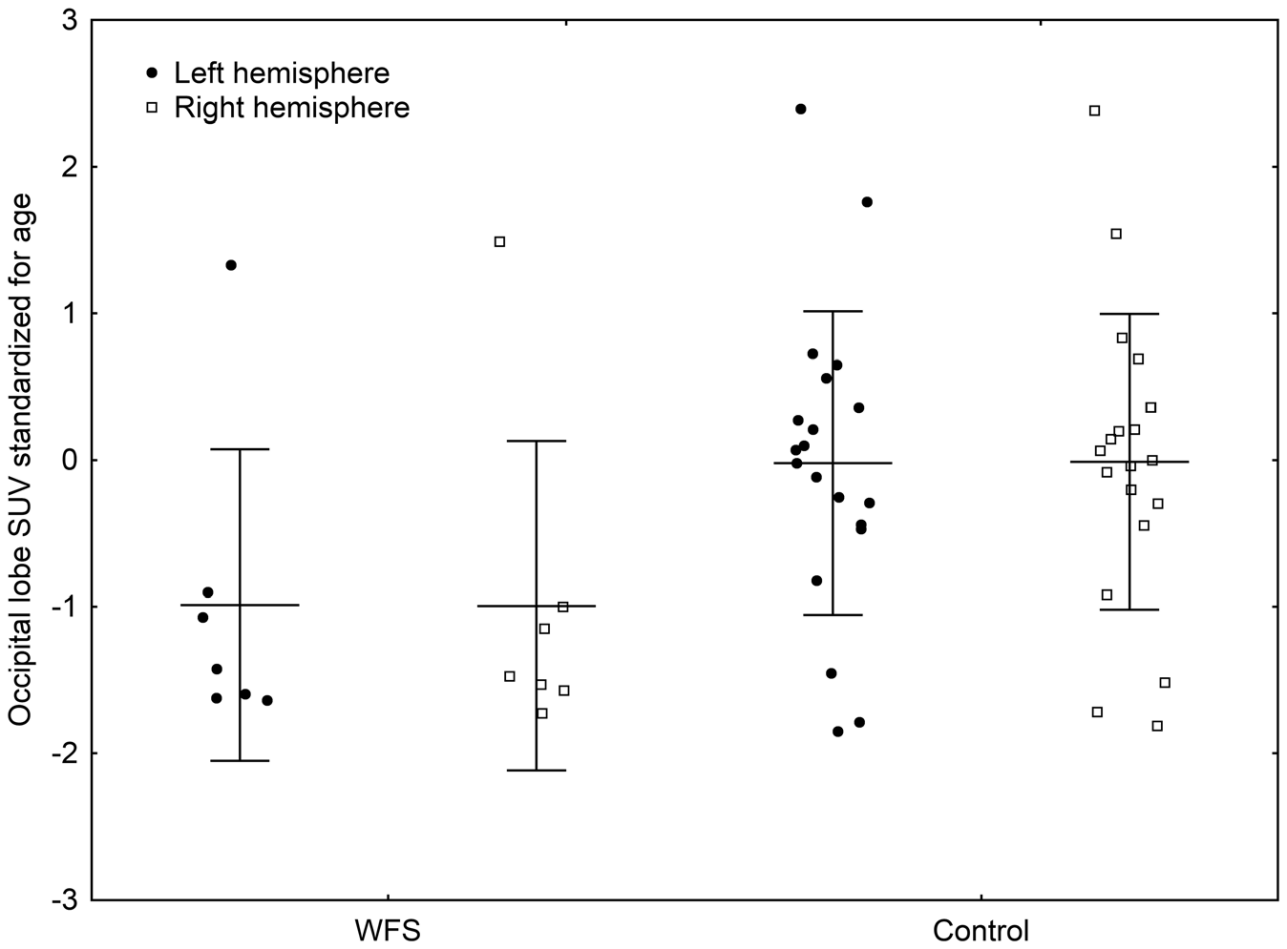
Mean SUV and SD values (standardized for age) for the right and left occipital lobes in WFS and control groups.

Hierarchical clustering showed similarities in glucose uptake patterns among the WFS patients, differing from those of the control group (Fig. 1). SUVs for all regions of the brain were lower in patients with WFS than in controls (S1 Table and Fig. 2), although the differences did not reach significance after adjustment for multiple hypothesis testing. After standardization, the differences persisted, with the WFS group showing SUVs on average 1 standard deviation lower than those of the control group, confirming that the effect was not due to differences in patient age or sex. We observed reduced SUVs in WFS patients as compared to the control group for the bilateral brain regions such as occipital lobe and cerebellum and the same tendency for cingulate, temporal lobe, parietal lobe, central region, basal ganglia and mesial temporal lobe (Table 3 and Fig. 3 and Fig. 4). However, the comparison of standardized SUVs between groups did not reach significance after adjustment for multiple hypothesis testing. In both groups, we observed no significant associations between raw or standardized SUVs of FDG uptake and age or sex (the latter comparison being only possible in controls).

**Table 3:** Differences in mean SUVs for selected main brain regions between WFS patients.

Brain region	Mean SUV±SD of WFS patients	Mean SUV±SD control group	p	Adjusted p
Frontal lobe	−0.63±0.77	0.01±1.04	0.156	0.156
Temporal lobe	−1.10±0.98	−0.15±1.10	0.057	0.085
Parietal lobe	−1.06±1.20	−0.08±1.08	0.058	0.085
Cingulate	−1.13±1.05	−0.15±1.12	0.056	0.085
Central region	−1.01±1.04	−0.09±1.06	0.060	0.085
Occipital lobe	−1.24±1.20	−0.13±1.05	0.028	0.085
Basal ganglia	−1.05±0.74	−0.20±1.07	0.066	0.085
Mesial temporal lobe	−1.06±0.82	−0.26±1.08	0.087	0.098
Cerebellum	−1.11±0.69	−0.204±1.00	0.036	0.085

and a control group (standardized bilateral data).

Brain MRI revealed small areas of atrophy in the brain stem and in the paraventricular white matter of both hemispheres only in one patient (WFS6) (Fig. 2). Patient WFS6 had areas of hiperintense signal in brain stem visible on T2-weighted and FLAIR MR images in sagittal and axial scans measured in three dimensions (AP = 3 mm, H = 7 mm, T = 15 mm).

In other WFS patients no brain stem, cerebellar or cerebral abnormalities were detected.

The MRI examinations also showed bilateral optic atrophy in 6 patients (WFS1-6) (Table 2). The optic nerves, chiasm and tracts were abnormally thin. The defect of optic nerves was more visible in the intracranial region (1–2 mm) than in the intraorbital (2–3 mm) by measuring the transverse diameter in axial, coronal and sagittal scans on T2-weighted MR images.

## Discussion

In this study we evaluated aspects of brain structure and function in pediatric patients with WFS compared to a control group by using PET-CT. We found differences in cerebral FDG uptake between patients with WFS and healthy children. The WFS patients in our study exhibited lower FDG uptake in the cerebellum and occipital lobe and also a tendency toward reduced SUVs for all other brain regions, but especially the cingulate, parietal and temporal lobes, central region, basal ganglia and mesial temporal lobe. Only patient WSF1 appears to be different from the other patients with WFS. This was a younger sister of patient WFS2 and had only mild neurological symptoms. One may speculate that this difference was related to the disease duration.

Interestingly, in those patients in whom brain MRI had been carried out about 1–2 years prior, no such advanced changes were detected apart from atrophy of optic nerves and multiple small lesions in the brain stem and in the paraventricular white matter of one patient. This may suggest that functional disturbances observed in patients with WFS can precede detectable structural brain changes. In addition, neurological examination showed cerebellar ataxia and gait abnormalities to be the main signs characteristic of our WFS patients, which were also the most common and earliest neurological signs in other patients with this syndrome [17], [18]. Other signs present in our patients such as nystagmus or hyporeflexia may also result from reduced function of the cerebellum, which was firmly confirmed in our PET-CT scans. Furthermore, in some of our patients, additional features like cognitive impairment; dysphagia and polyneuropathy were also present, which could be related to the reduced activity in the basal ganglia, central region, occipital lobe, cingulate and/or temporal lobe as detected by PET-CT. However, a small number of studies concerning normal patterns of brain FDG uptake in children is problematic due to a lack of public available comprehensive database [19], [20].

In addition to the characteristic locations of the brain changes visible on PET-CT in our WFS patients, the occurrence of these changes in relationship to patient age should be also noted. The ages in the study group ranged from 10 to 16 years and averaged about 13 years. Previous descriptions of neurological disorders in WFS were mainly based on studies of adult patients, which explains the advanced lesions reported in the prior neuroimaging studies [21], [22], [23]. A study reporting post-mortem findings in a patient with WFS documented, besides severe degeneration of the optic nerves, severe loss of neurons from lateral geniculate nuclei, pons, hypothalamic nuclei, optic radiations, hippocampal fornices and deep cerebral white matter, and axonal dystrophy in the pontocerebral tracts [11]. Other authors have emphasized the correlation between the presence of neurological defects such as loss of vision, nystagmus, gait disturbances, hearing loss, depression, anosmia, and epilepsy in WFS and the results of neuropathological post-mortem examinations [24], [25]. However, it is worth noting that recent results of Hershey et al. [26] suggest possible involvement of two pathological processes contributing to the brain abnormalities in WFS, one that is neurodevelopmental in nature and the other neurodegenerative. In that study, the abnormalities that correlated with patient age were evaluated at the earliest stage of disease using both clinical and cognitive testing, and neuroimaging (MRI) as well. Comparison of the results of the neuroimaging studies in children with WFS versus type 1 diabetic children and healthy children showed the presence of different abnormalities in children with WFS, including smaller intracranial volume and disorders of microstructural integrity in the brain stem, cerebellum and optic radiations. The abnormalities were detected even in the youngest patients with very mild clinical symptoms. This is a new potential insight into a WFS disease process, which also draws attention to the need for neuroimaging studies in the early stages of diagnosis of WFS.

Indeed, our study was conducted in children with WFS at an early stage of the disease, before the development of a full range of neurological disorders. Our study is also the first to use PET-CT to describe regional differences in brain glucose metabolism in children with WFS, including reduced regional brain activity in these patients.

There are some limitations to our study. First, because of the low frequency of WFS in the European population we do not have a large group of patients with this syndrome.

In addition, insulin-dependent diabetes present in patients with WFS can influence the observed brain changes. There are also no published studies available reporting results of PET-CT in children with type 1 diabetes, which could be used to define correlations between brain SUVs and HbA1c level, glycemia level or diabetes duration. To date, FDG-PET-CT has been very useful for detection of regional differences in cerebral glucose transport in patients with symptomatic (‘aware’) versus asymptomatic (‘unaware’) hypoglycemia in type 1 diabetes [27]. PET-CT analyses in type 1 diabetic patients have focused on identifying cerebral correlates of hypoglycemia unawareness. According to one study, the regions of interest are the amygdala and frontal cortex [27], whereas results of another study suggest a subthalamic region can exhibit significantly different behavior between aware and unaware patient groups [28]. During hypoglycemia, brain glucose content was observed to fall in both aware and unaware patients with type 1 diabetes, with a relative increase in tracer uptake in prefrontal cortical regions [29]. We can consider whether a glucose level at the time of examination in our patients may influence the results. The WFS patients were normoglycemic at the time of evaluation and we did not observe any consistent impact on SUV levels. It should be also emphasized that our observations concern different areas of the brain than for patients with type 1 diabetes with hypoglycemia.

Finally, more precise neurological evaluation of WFS patients is needed in order to better assess associations between clinical signs and the results of the brain PET-CT study. In the future, PET-CT could become an important tool for evaluating the effectiveness of potential drugs in patients with WFS.

## Supporting Information

S1 Table Mean SUVs for all brain regions in patients with WFS and a control group (nonstandardized data; L, left side of the brain; R, right side of the brain). Regions with differences significant in unadjusted comparisons are marked in bold.
(DOCX)
