# Supplementary material for: Central Nervous System PET-CT Imaging Reveals Regional Impairments in Pediatric Patients with Wolfram Syndrome
Source: PLoS One. 2014 Dec 26;9(12):e115605. doi: 10.1371/journal.pone.0115605 (PMC4277290; doi:10.1371/journal.pone.0115605)
Supplement: S1 Table — Mean SUVs for all brain regions in patients with WFS and a control group (nonstandardized data; L, left side of the brain; R, right side of the brain). Regions with differences significant in unadjusted comparisons are marked in bold. (DOCX) [file pone.0115605.s001.docx]

Table S1. Mean SUVs for all brain regions in patients with WFS and a control group (nonstandardized data; L, left side of the brain; R, right side of the brain). Regions with differences significant in unadjusted comparisons are marked in bold.

|  | SUV in WFS patients [mean±SD] | SUV in Controls [mean±SD] | p | adjusted p | Age-standardized SUV in WFS patients [mean±SD] | Age-standardized SUV in Controls [mean±SD] | p | adjusted p |
| --- | --- | --- | --- | --- | --- | --- | --- | --- |
| Amygdala L-mean SUV | 3.69±0.81 | 4.51±1.23 | 0.116 | 0.140 | -0.64±0.69 | -0.02±1.19 | 0.207 | 0.225 |
| Amygdala R-mean SUV | 3.49±0.70 | 4.26±1.22 | 0.131 | 0.151 | -0.60±0.63 | 0.02±1.20 | 0.209 | 0.225 |
| Angular gyrus L-mean SUV | **6.78±2.54** | **9.21±2.53** | **0.039** | 0.119 | -0.88±1.01 | -0.02±1.05 | 0.071 | 0.186 |
| Angular gyrus R-mean SUV | **6.48±2.37** | **8.82±2.41** | **0.036** | 0.119 | -0.89±0.93 | -0.02±1.03 | 0.059 | 0.186 |
| Anterior cingulate L-mean SUV | 6.07±1.74 | 7.43±1.91 | 0.112 | 0.140 | -0.61±0.91 | -0.02±1.11 | 0.220 | 0.233 |
| Anterior cingulate R-mean SUV | 5.83±1.56 | 7.00±1.75 | 0.129 | 0.150 | -0.58±0.89 | -0.01±1.07 | 0.222 | 0.234 |
| Basal ganglia L-mean SUV | 5.99±1.71 | 7.84±2.38 | 0.071 | 0.119 | -0.74±0.69 | -0.03±1.07 | 0.117 | 0.186 |
| Basal ganglia R-mean SUV | 5.96±1.72 | 8.01±2.56 | 0.062 | 0.119 | -0.76±0.64 | -0.03±1.06 | 0.100 | 0.186 |
| Calcarine fissure L-mean SUV | **6.24±2.61** | **9.83±2.59** | **0.004** | 0.119 | **-1.34±0.93** | **-0.02±1.10** | **0.009** | 0.186 |
| Calcarine fissure R-mean SUV | **6.35±2.62** | **9.19±2.12** | **0.008** | 0.119 | **-1.34±1.13** | **0.02±1.07** | **0.008** | 0.186 |
| Caudate nucleus L-mean SUV | 5.38±1.87 | 6.67±1.92 | 0.138 | 0.157 | -0.61±1.06 | -0.08±1.19 | 0.307 | 0.314 |
| Caudate nucleus R-mean SUV | 5.35±1.75 | 7.04±2.23 | 0.082 | 0.119 | -0.72±0.82 | -0.05±1.12 | 0.165 | 0.200 |
| Central region L-mean SUV | 6.29±2.12 | 8.19±2.12 | 0.053 | 0.119 | -0.83±0.95 | -0.01±1.01 | 0.076 | 0.186 |
| Central region R-mean SUV | 6.24±2.05 | 8.00±2.12 | 0.069 | 0.119 | -0.76±0.92 | -0.01±1.02 | 0.103 | 0.186 |
| Cerebellum L-mean SUV | **4.77±1.35** | **6.43±1.81** | **0.037** | 0.119 | -0.80±0.68 | 0.01±1.05 | 0.070 | 0.186 |
| Cerebellum R-mean SUV | **4.51±1.15** | **6.14±1.80** | **0.036** | 0.119 | -0.88±0.69 | 0.00±1.06 | 0.052 | 0.186 |
| Cerebelum-10 L-mean SUV | 3.43±1.13 | 4.20±1.20 | 0.151 | 0.166 | -0.84±0.65 | 0.00±1.04 | 0.057 | 0.186 |
| Cerebelum-10 R-mean SUV | 3.40±1.17 | 4.01±1.14 | 0.237 | 0.246 | -0.87±0.59 | 0.00±1.06 | 0.052 | 0.186 |
| Cerebelum-3 L-mean SUV | 4.61±1.05 | 5.33±1.57 | 0.273 | 0.279 | -0.76±0.89 | 0.04±1.19 | 0.116 | 0.186 |
| Cerebelum-3 R-mean SUV | 4.62±1.40 | 5.33±1.43 | 0.263 | 0.271 | -0.66±0.98 | 0.01±1.13 | 0.177 | 0.205 |
| Cerebelum-4-5 L-mean SUV | 5.22±1.50 | 6.27±1.74 | 0.170 | 0.182 | -0.43±0.62 | 0.03±1.08 | 0.289 | 0.297 |
| Cerebelum-4-5 R-mean SUV | 5.37±1.57 | 6.80±1.89 | 0.085 | 0.119 | -0.47±0.95 | -0.01±1.03 | 0.315 | 0.319 |
| Cerebelum-6 L-mean SUV | **5.51±1.62** | **7.44±2.16** | **0.042** | 0.119 | -0.58±0.80 | 0.01±1.03 | 0.189 | 0.213 |
| Cerebelum-6 R-mean SUV | **5.32±1.42** | **7.12±2.05** | **0.042** | 0.119 | -0.72±0.78 | -0.01±1.04 | 0.114 | 0.186 |
| Cerebelum-7b L-mean SUV | **4.56±1.22** | **6.22±1.78** | **0.032** | 0.119 | **-0.87±0.67** | **0.01±1.03** | **0.047** | 0.186 |
| Cerebelum-7b R-mean SUV | **4.29±1.12** | **6.20±2.00** | **0.025** | 0.119 | **-0.85±0.61** | **0.01±1.04** | **0.049** | 0.186 |
| Cerebelum-8 L-mean SUV | **4.65±1.16** | **6.15±1.72** | **0.043** | 0.119 | -0.89±0.70 | -0.03±1.08 | 0.062 | 0.186 |
| Cerebelum-8 R-mean SUV | **4.55±1.12** | **6.14±1.75** | **0.035** | 0.119 | **-0.92±0.56** | **-0.02±1.04** | **0.041** | 0.186 |
| Cerebelum-9 L-mean SUV | 4.61±1.04 | 5.96±1.71 | 0.063 | 0.119 | -0.84±0.63 | 0.00±1.06 | 0.062 | 0.186 |
| Cerebelum-9 R-mean SUV | **4.65±1.09** | **6.16±1.78** | **0.045** | 0.119 | **-0.88±0.58** | **0.01±1.05** | **0.046** | 0.186 |
| Cerebelum-crus 2 L-mean SUV | **4.59±1.36** | **6.32±1.87** | **0.035** | 0.119 | -0.76±0.56 | 0.02±1.11 | 0.087 | 0.186 |
| Cerebelum-crus1 L-mean SUV | **4.50±1.45** | **6.51±1.88** | **0.017** | 0.119 | -0.83±0.56 | 0.02±1.08 | 0.060 | 0.186 |
| Cerebelum-crus1 R-mean SUV | **4.11±1.06** | **5.94±1.76** | **0.016** | 0.119 | -0.89±0.74 | -0.02±1.07 | 0.059 | 0.186 |
| Cerebelum-crus2 R-mean SUV | **3.93±1.01** | **5.73±1.79** | **0.019** | 0.119 | **-1.04±0.71** | **0.01±1.08** | **0.025** | 0.186 |
| Cingulate and par. L-mean SUV | 6.35±2.04 | 8.10±2.10 | 0.068 | 0.119 | **-1.00±0.56** | **0.00±1.05** | **0.025** | 0.186 |
| Cingulate and par. R-mean SUV | **6.06±1.76** | **7.80±1.92** | **0.046** | 0.119 | **-0.98±0.52** | **0.01±1.04** | **0.025** | 0.186 |
| Cuneus L-mean SUV | 6.76±2.97 | 8.77±2.18 | 0.069 | 0.119 | -0.75±0.92 | -0.03±1.06 | 0.121 | 0.186 |
| Cuneus R-mean SUV | **6.87±3.08** | **9.20±2.34** | **0.047** | 0.119 | -0.83±0.85 | -0.03±1.07 | 0.087 | 0.186 |
| Frontal lobe L-mean SUV | 6.54±1.95 | 8.30±2.33 | 0.086 | 0.119 | -0.86±1.30 | -0.01±1.01 | 0.085 | 0.186 |
| Frontal lobe R-mean SUV | 6.59±1.90 | 8.29±2.31 | 0.093 | 0.124 | -0.92±1.30 | -0.01±1.01 | 0.069 | 0.186 |
| Fusiform gyrus L-mean SUV | 5.65±1.76 | 7.15±1.74 | 0.062 | 0.119 | -0.69±0.80 | -0.02±1.04 | 0.137 | 0.190 |
| Fusiform gyrus R-mean SUV | 5.65±1.66 | 7.10±1.63 | 0.055 | 0.119 | -0.67±0.79 | -0.03±1.05 | 0.151 | 0.195 |
| Gyrus rectus L-mean SUV | 6.52±1.50 | 8.22±2.21 | 0.073 | 0.119 | -0.78±1.04 | -0.05±1.12 | 0.140 | 0.190 |
| Gyrus rectus R-mean SUV | 6.63±1.50 | 8.23±2.17 | 0.085 | 0.119 | -0.81±0.98 | -0.03±1.06 | 0.099 | 0.186 |
| Heschl gyrus L-mean SUV | 7.05±2.03 | 9.26±2.57 | 0.051 | 0.119 | -0.70±0.62 | -0.02±1.04 | 0.117 | 0.186 |
| Heschl gyrus R-mean SUV | **6.95±2.03** | **9.29±2.72** | **0.049** | 0.119 | -0.68±0.65 | -0.01±1.01 | 0.112 | 0.186 |
| Hippocampus L-mean SUV | 3.65±0.77 | 4.54±1.20 | 0.079 | 0.119 | -0.80±0.66 | -0.02±1.05 | 0.080 | 0.186 |
| Hippocampus R-mean SUV | 3.64±0.77 | 4.34±1.09 | 0.128 | 0.150 | -0.78±0.73 | -0.04±1.14 | 0.123 | 0.187 |
| inferior frontal gyrus L-mean SUV | 6.48±1.92 | 8.41±2.36 | 0.063 | 0.119 | -0.76±0.84 | -0.01±1.01 | 0.091 | 0.186 |
| inferior frontal gyrus L-mean SUV | 6.45±2.14 | 8.49±2.47 | 0.064 | 0.119 | -0.71±0.62 | -0.05±1.12 | 0.155 | 0.195 |
| inferior frontal gyrus L-mean SUV | 6.53±1.93 | 8.58±2.39 | 0.052 | 0.119 | -0.62±0.70 | -0.02±1.11 | 0.201 | 0.220 |
| inferior frontal gyrus R-mean SUV | 6.76±1.84 | 8.72±2.47 | 0.067 | 0.119 | -0.80±0.72 | -0.01±1.02 | 0.072 | 0.186 |
| inferior frontal gyrus R-mean SUV | 6.24±1.84 | 8.06±2.24 | 0.065 | 0.119 | -0.76±0.77 | -0.02±1.03 | 0.097 | 0.186 |
| inferior frontal gyrus R-mean SUV | 6.02±1.61 | 7.71±2.23 | 0.078 | 0.119 | -0.74±0.67 | -0.03±1.06 | 0.112 | 0.186 |
| Inferior occipital gyrus L-mean SUV | **6.19±2.27** | **8.36±2.24** | **0.037** | 0.119 | -0.74±0.76 | -0.02±1.04 | 0.107 | 0.186 |
| Inferior occipital gyrus R-mean SUV | **6.10±2.23** | **8.56±2.29** | **0.021** | 0.119 | -0.70±0.69 | -0.01±1.04 | 0.120 | 0.186 |
| Inferior parietal bu. L-mean SUV | **6.87±2.56** | **9.17±2.41** | **0.042** | 0.119 | -0.90±0.99 | -0.02±1.03 | 0.061 | 0.186 |
| Inferior parietal bu. R-mean SUV | 6.96±2.71 | 9.07±2.28 | 0.056 | 0.119 | **-0.99±0.99** | **-0.01±1.06** | **0.043** | 0.186 |
| Inferior temporal gyrus L-mean SUV | 5.78±1.67 | 7.37±2.01 | 0.073 | 0.119 | -0.85±1.06 | -0.02±1.08 | 0.091 | 0.186 |
| Inferior temporal gyrus R-mean SUV | **5.66±1.61** | **7.41±1.99** | **0.046** | 0.119 | -0.81±1.16 | -0.03±1.08 | 0.119 | 0.186 |
| Insula L-mean SUV | 6.13±1.55 | 7.74±2.16 | 0.082 | 0.119 | -0.73±0.77 | -0.03±1.05 | 0.118 | 0.186 |
| Insula R-mean SUV | 6.31±1.75 | 8.04±2.24 | 0.077 | 0.119 | -0.83±0.71 | -0.02±1.05 | 0.071 | 0.186 |
| Lentricular nucleus L-mean SUV | 6.86±2.05 | 9.27±3.07 | 0.066 | 0.119 | -0.70±0.64 | -0.02±1.03 | 0.118 | 0.186 |
| Lentricular nucleus L-mean SUV | 5.42±1.20 | 6.73±1.90 | 0.102 | 0.129 | -0.71±0.74 | -0.03±1.06 | 0.132 | 0.190 |
| Lentricular nucleus R-mean SUV | 6.61±1.93 | 8.90±2.94 | 0.067 | 0.119 | -0.75±0.62 | -0.01±1.03 | 0.087 | 0.186 |
| Lentricular nucleus R-mean SUV | 5.98±1.80 | 8.02±2.66 | 0.073 | 0.119 | -0.66±0.54 | 0.01±1.10 | 0.134 | 0.190 |
| Lingual gyrus L-mean SUV | **6.31±2.50** | **9.04±2.57** | **0.022** | 0.119 | -0.76±0.58 | 0.01±1.07 | 0.084 | 0.186 |
| Lingual gyrus R-mean SUV | **6.21±2.51** | **8.84±2.42** | **0.021** | 0.119 | -0.72±0.74 | -0.03±1.15 | 0.155 | 0.195 |
| Mesial temporal lobe L-mean SUV | 3.99±0.91 | 4.93±1.24 | 0.077 | 0.119 | **-1.00±0.90** | **-0.02±1.06** | **0.039** | 0.186 |
| Mesial temporal lobe R-mean SUV | 4.07±0.96 | 4.95±1.23 | 0.099 | 0.128 | **-1.05±0.96** | **0.00±1.00** | **0.024** | 0.186 |
| Middle cingulate a. L-mean SUV | 6.66±2.23 | 8.50±2.23 | 0.072 | 0.119 | -0.73±0.73 | -0.05±1.12 | 0.152 | 0.195 |
| Middle cingulate a. R-mean SUV | **6.37±1.90** | **8.38±2.08** | **0.034** | 0.119 | -0.68±0.75 | -0.02±1.10 | 0.157 | 0.195 |
| middle frontal gyrus L-mean SUV | 6.38±1.71 | 8.42±2.48 | 0.056 | 0.119 | -0.90±0.84 | -0.03±1.07 | 0.064 | 0.186 |
| middle frontal gyrus L-mean SUV | 7.01±2.18 | 9.00±2.60 | 0.081 | 0.119 | -0.75±0.94 | -0.02±1.04 | 0.117 | 0.186 |
| middle frontal gyrus R-mean SUV | 6.35±1.70 | 7.97±2.29 | 0.101 | 0.129 | -0.77±0.64 | -0.02±1.03 | 0.085 | 0.186 |
| middle frontal gyrus R-mean SUV | 7.16±2.35 | 9.17±2.66 | 0.090 | 0.122 | -0.70±0.82 | -0.03±1.07 | 0.145 | 0.190 |
| Middle occipital gyrus L-mean SUV | **6.54±2.57** | **8.82±2.42** | **0.044** | 0.119 | -0.69±0.87 | -0.03±1.07 | 0.158 | 0.195 |
| Middle occipital gyrus R-mean SUV | **6.48±2.58** | **8.65±2.20** | **0.042** | 0.119 | -0.63±0.70 | -0.02±1.07 | 0.175 | 0.204 |
| Middle temporal gyrus L-mean SUV | 6.31±1.86 | 8.04±2.12 | 0.067 | 0.119 | -0.88±1.03 | -0.01±1.00 | 0.060 | 0.186 |
| Middle temporal gyrus R-mean SUV | 6.13±2.03 | 8.00±2.12 | 0.054 | 0.119 | -0.89±1.19 | 0.00±1.03 | 0.072 | 0.186 |
| Occipital lobe L-mean SUV | **6.28±2.47** | **8.64±2.23** | **0.027** | 0.119 | -0.74±0.84 | -0.03±1.07 | 0.128 | 0.190 |
| Occipital lobe R-mean SUV | **6.27±2.44** | **8.47±2.08** | **0.029** | 0.119 | -0.79±0.90 | -0.03±1.07 | 0.107 | 0.186 |
| Olfactory cortex L-mean SUV | 4.86±1.08 | 6.03±1.53 | 0.076 | 0.119 | **-0.99±1.06** | **-0.02±1.03** | **0.044** | 0.186 |
| Olfactory cortex R-mean SUV | 4.84±1.17 | 6.16±1.58 | 0.054 | 0.119 | **-0.99±1.12** | **-0.01±1.01** | **0.041** | 0.186 |
| Paracentral lobule L-mean SUV | 5.85±1.81 | 7.08±1.92 | 0.154 | 0.166 | -0.71±0.71 | -0.02±1.07 | 0.130 | 0.190 |
| Paracentral lobule R-mean SUV | 5.66±1.72 | 7.01±1.91 | 0.112 | 0.140 | -0.79±0.69 | -0.02±1.02 | 0.077 | 0.186 |
| Parahippocampal. L-mean SUV | 4.38±1.09 | 5.40±1.29 | 0.074 | 0.119 | -0.58±0.95 | -0.02±1.05 | 0.227 | 0.236 |
| Parahippocampal. R-mean SUV | 4.57±1.21 | 5.61±1.37 | 0.087 | 0.119 | -0.65±0.86 | -0.01±1.00 | 0.144 | 0.190 |
| Parietal lobe L-mean SUV | 6.53±2.34 | 8.45±2.17 | 0.059 | 0.119 | -0.75±0.84 | -0.05±1.11 | 0.145 | 0.190 |
| Parietal lobe R-mean SUV | 6.34±2.34 | 8.19±2.03 | 0.057 | 0.119 | -0.72±0.84 | -0.03±1.08 | 0.139 | 0.190 |
| Postcentral gyrus L-mean SUV | **6.04±2.08** | **7.97±2.01** | **0.040** | 0.119 | -0.80±1.06 | -0.02±1.04 | 0.100 | 0.186 |
| Postcentral gyrus R-mean SUV | 5.95±1.94 | 7.70±2.01 | 0.057 | 0.119 | -0.83±1.10 | -0.01±1.01 | 0.081 | 0.186 |
| Posterior cingulate L-mean SUV | **5.84±2.21** | **8.45±2.36** | **0.017** | 0.119 | -0.90±0.97 | -0.01±1.00 | 0.052 | 0.186 |
| Posterior cingulate R-mean SUV | **4.94±1.66** | **7.13±1.91** | **0.013** | 0.119 | -0.80±0.92 | -0.01±1.01 | 0.084 | 0.186 |
| precentral gyrus L-mean SUV | 6.55±2.28 | 8.43±2.25 | 0.070 | 0.119 | **-1.06±0.84** | **-0.01±1.07** | **0.028** | 0.186 |
| precentral gyrus R - mean SUV | 6.53±2.28 | 8.34±2.29 | 0.084 | 0.119 | **-1.13±0.76** | **0.01±1.14** | **0.022** | 0.186 |
| Precuneus L-mean SUV | 6.65±2.28 | 8.23±2.17 | 0.114 | 0.140 | -0.77±0.98 | -0.02±1.03 | 0.105 | 0.186 |
| Precuneus R-mean SUV | 6.65±2.26 | 8.26±2.04 | 0.093 | 0.124 | -0.72±0.96 | -0.02±1.03 | 0.128 | 0.190 |
| Rolandic operculum L-mean SUV | 6.37±1.76 | 8.19±2.20 | 0.060 | 0.119 | -0.66±1.03 | -0.02±1.04 | 0.174 | 0.204 |
| Rolandic operculum R-mean SUV | 6.31±1.77 | 8.00±2.09 | 0.069 | 0.119 | -0.73±1.06 | -0.01±1.00 | 0.120 | 0.186 |
| superior frontal gyrus L-mean SUV | 6.41±1.97 | 7.97±2.24 | 0.115 | 0.140 | -0.76±0.77 | -0.01±1.02 | 0.089 | 0.186 |
| superior frontal gyrus L-mean SUV | 6.54±1.80 | 8.29±2.47 | 0.099 | 0.128 | -0.73±0.81 | -0.01±1.02 | 0.102 | 0.186 |
| Superior frontal gyrus L-mean SUV | 6.62±1.83 | 8.47±2.32 | 0.067 | 0.119 | -0.65±0.71 | -0.02±1.06 | 0.163 | 0.200 |
| Superior frontal gyrus L-mean SUV | 6.40±1.78 | 7.92±2.26 | 0.121 | 0.144 | -0.63±0.86 | -0.03±1.07 | 0.194 | 0.214 |
| superior frontal gyrus R-mean SUV | 6.58±1.68 | 8.37±2.36 | 0.078 | 0.119 | -0.73±0.76 | -0.02±1.05 | 0.115 | 0.186 |
| Superior frontal gyrus R-mean SUV | 6.36±1.59 | 8.05±2.12 | 0.066 | 0.119 | -0.70±0.69 | -0.03±1.08 | 0.142 | 0.190 |
| superior frontal gyrus R-mean SUV | 6.71±2.06 | 8.17±2.25 | 0.144 | 0.161 | -0.62±0.75 | -0.02±1.03 | 0.173 | 0.204 |
| Superior frontal gyrus R-mean SUV | 6.37±1.57 | 7.95±2.19 | 0.094 | 0.124 | -0.59±0.86 | -0.03±1.07 | 0.226 | 0.236 |
| Superior occipital L-mean SUV | 6.30±2.78 | 8.17±1.93 | 0.060 | 0.119 | -0.67±0.67 | -0.02±1.04 | 0.135 | 0.190 |
| Superior occipital R-mean SUV | 6.54±2.83 | 8.33±2.05 | 0.083 | 0.119 | -0.73±0.74 | -0.01±1.05 | 0.110 | 0.186 |
| Superior parietal gyrus L-mean SUV | **5.82±2.25** | **7.57±1.81** | **0.049** | 0.119 | -0.89±1.44 | -0.01±1.00 | 0.083 | 0.186 |
| Superior parietal gyrus R-mean SUV | 5.41±2.28 | 6.87±1.62 | 0.076 | 0.119 | -0.81±1.36 | -0.01±1.00 | 0.111 | 0.186 |
| Superior temporal L-mean SUV | 6.31±1.95 | 8.14±2.07 | 0.053 | 0.119 | -0.89±1.23 | 0.00±1.00 | 0.068 | 0.186 |
| Superior temporal R-mean SUV | 6.13±1.86 | 7.81±1.92 | 0.055 | 0.119 | -0.91±1.35 | 0.00±0.98 | 0.067 | 0.186 |
| Supplementary m.L-mean SUV | 6.73±2.34 | 8.30±2.35 | 0.142 | 0.159 | -0.81±0.87 | -0.02±1.04 | 0.086 | 0.186 |
| Supplementary m.R-mean SUV | 6.66±1.99 | 8.18±2.31 | 0.134 | 0.153 | -0.80±0.88 | -0.02±1.04 | 0.092 | 0.186 |
| Supramarginal gyrus L-mean SUV | **6.44±2.14** | **8.42±2.18** | **0.049** | 0.119 | -0.62±0.93 | -0.01±1.02 | 0.183 | 0.209 |
| Supramarginal gyrus R-mean SUV | **6.30±2.36** | **8.37±2.17** | **0.043** | 0.119 | -0.60±0.83 | -0.02±1.04 | 0.194 | 0.214 |
| Temporal lobe L-mean SUV | 6.17±1.82 | 7.89±2.08 | 0.064 | 0.119 | -0.81±0.96 | -0.02±1.05 | 0.089 | 0.186 |
| Temporal lobe R-mean SUV | **6.00±1.85** | **7.79±2.03** | **0.050** | 0.119 | -0.86±1.05 | -0.01±1.02 | 0.072 | 0.186 |
| Temporal pole: mi. L-mean SUV | 4.59±1.00 | 5.73±1.45 | 0.067 | 0.119 | -0.76±0.82 | -0.03±1.06 | 0.113 | 0.186 |
| Temporal pole: mi. R-mean SUV | 4.28±0.77 | 5.21±1.23 | 0.073 | 0.119 | -0.81±0.83 | -0.03±1.06 | 0.090 | 0.186 |
| Temporal pole: su. L-mean SUV | 4.72±1.21 | 5.58±1.63 | 0.214 | 0.225 | -0.75±0.60 | -0.02±1.09 | 0.106 | 0.186 |
| Temporal pole: su. R-mean SUV | 4.28±0.96 | 5.00±1.28 | 0.186 | 0.198 | -0.75±0.51 | 0.03±1.09 | 0.087 | 0.186 |
| Thalamus L-mean SUV | **5.44±1.62** | **7.29±2.16** | **0.050** | 0.119 | -0.55±0.64 | 0.04±1.08 | 0.190 | 0.213 |
| Thalamus R-mean SUV | **5.45±1.65** | **7.46±2.21** | **0.038** | 0.119 | -0.57±0.65 | 0.03±1.05 | 0.171 | 0.204 |
| Vermis Mean | 5.06±1.26 | 6.34±1.92 | 0.117 | 0.141 | -0.81±0.76 | -0.03±1.07 | 0.090 | 0.186 |
| Vermis-10 B mean SUV | 4.47±0.98 | 5.06±1.83 | 0.435 | 0.435 | -0.86±0.74 | -0.03±1.05 | 0.066 | 0.186 |
| Vermis-10 B mean SUV | 4.47±0.98 | 5.06±1.83 | 0.435 | 0.435 | -0.65±0.59 | 0.05±1.12 | 0.132 | 0.190 |
| Vermis-1-2 B mean SUV | 5.00±0.90 | 5.72±1.98 | 0.365 | 0.371 | -0.27±0.55 | 0.08±1.20 | 0.464 | 0.464 |
| Vermis-3 B mean SUV | 4.36±0.96 | 5.13±1.53 | 0.228 | 0.238 | -0.32±0.48 | 0.07±1.19 | 0.402 | 0.405 |
| Vermis-4-5 B mean SUV | 4.97±1.35 | 6.17±1.84 | 0.129 | 0.150 | -0.51±0.58 | 0.04±1.09 | 0.219 | 0.233 |
| Vermis-6 B mean SUV | **4.88±1.49** | **6.88±2.30** | **0.043** | 0.119 | -0.64±0.67 | 0.04±1.09 | 0.141 | 0.190 |
| Vermis-7 B mean SUV | **4.87±1.71** | **6.58±1.84** | **0.041** | 0.119 | **-0.89±0.60** | **0.04±1.10** | **0.044** | 0.186 |
| Vermis-8 B mean SUV | 5.78±1.59 | 7.59±2.31 | 0.069 | 0.119 | **-0.92±0.86** | **0.03±1.06** | **0.044** | 0.186 |
| Vermis-9 B mean SUV | 6.16±1.49 | 7.57±2.35 | 0.154 | 0.166 | -0.75±0.64 | 0.00±1.07 | 0.095 | 0.186 |
| Vermis-9 B mean SUV | 6.16±1.49 | 7.57±2.35 | 0.154 | 0.166 | -0.58±0.56 | 0.05±1.15 | 0.181 | 0.208 |
